# Anisotropic PDMS/Alumina/Carbon Fiber Composites with a High Thermal Conductivity and an Electromagnetic Interference Shielding Performance

**DOI:** 10.3390/ma15228078

**Published:** 2022-11-15

**Authors:** Xi Zhang, Jianan Song, Jiajia Meng, Kan Zhang

**Affiliations:** Research School of Polymeric Materials, School of Materials Science & Engineering, Jiangsu University, Zhenjiang 212000, China

**Keywords:** thermal conductivity, electromagnetic interference shielding, polydimethylsiloxane, carbon fiber, alumina

## Abstract

The development of polymer-based composites with a high thermal conductivity and electromagnetic interference (EMI) shielding performance is crucial to the application of polymer-based composites in electronic equipment. Herein, a novel strategy combining ice-templated assembly and stress-induced orientation was proposed to prepare polydimethylsiloxane (PDMS)/alumina/carbon fiber (CF) composites. CF in the composites exhibited a highly oriented structure in the horizontal direction. Alumina was connected to the CF, promoting the formation of thermal conductive pathways in both the horizontal and vertical directions. As the CF content was 27.5 vol% and the alumina content was 14.0 vol%, the PDMS/alumina/CF composite had high thermal conductivities in the horizontal and vertical directions, which were 8.44 and 2.34 W/(m·K), respectively. The thermal conductivity in the horizontal direction was 40.2 times higher than that of PDMS and 5.0 times higher than that of the composite with a randomly distributed filler. The significant enhancement of the thermal conductivity was attributed to the oriented structure of the CF and the bridging effect of alumina. The PDMS/alumina/CF composite exhibited an excellent EMI shielding effectiveness of 40.8 dB which was 2.4 times higher than that of the composite with a randomly distributed filler. The PDMS/alumina/CF composite also exhibited a low reflectivity of the electromagnetic waves. This work could provide a guide for the research of polymer-based composites with a high thermal conductivity and an EMI shielding performance.

## 1. Introduction

In the wake of the developments in 5th generation (5G) mobile communication technology, more and more requirements are demanded in polymer-based composites [1]. For one thing, the microminiaturization and high power density of 5G electronic equipments lead to the increase in demand for heat dissipation [2,3]. For another, the electromagnetic interference (EMI) of the running equipment affects the reliability and stability of electronic equipment [4,5,6]. The development of EMI shielding materials is utterly urgent. The research of polymer-based composites with a high thermal conductivity and EMI shielding performance has become a hot spot [7].

In order to improve the thermal conductivity and EMI shielding performance of polymer-based composites, conductive fillers are usually added into the polymer matrix [8]. Metal powders and carbon-based fillers are common additives [9,10]. However, the addition of metal powders could lead to the poor processability, the high density and the poor mechanical properties of polymer composites. Carbon-based fillers have been regarded as good candidates to prepare polymer-based composites with a high thermal conductivity and EMI shielding performance, due to the many advantages of carbon-based fillers, such as a high electrical conductivity, a high thermal conductivity, excellent mechanical properties and they are lightweight [11,12]. For example, Lu et al. [13] improved the EMI shielding effectiveness of the ethylene propylene diene monomer rubber composite to 35 dB by adding 8 wt% graphene nanoplatelets. The thermal conductivity increased to 0.79 W/(m·K) at the same time. Wen et al. [14] used nickel coated graphite and short-cut carbon fibre to improve the EMI shielding performance of the polyvinyl butyral composites. Nah et al. [15] prepared carbon nanotubes/polyurethane composites through a phase separation strategy. With a filler content of 10 wt%, the composite had a remarkable EMI shielding effectiveness of 42.5 dB and a high thermal conductivity of 0.51 W/(m·K).

According to previous studies, the continuous structure of fillers is favorable to the improvement of the thermal conductivity and EMI shielding performance of polymer composites [16,17,18]. The continuous structure of fillers endowed the composites with the low conductive percolation threshold. Many approaches have been developed to construct the continuous structure of the fillers, such as the foaming method [19], layer-by-layer assembly [20,21], ice-templated assembly [22,23], vacuum-assisted filtration [24] and pre-constructed aerogel method [25,26], and so on. For example, Ren et al. [26] utilized the pre-constructed graphene nanosheets/carbon nanotubes framework to improve the thermal conductivity and EMI shielding performance of the composites. Feng et al. [27] fabricated interconnected carbon scaffolds and then impregnated the scaffolds with epoxy resin in order to reach the high thermal conductivity and the EMI shielding effectiveness. However, the low degree of orientation hindered the performance improvement of the composites. The layer-by-layer assembly and the vacuum-assisted filtration methods could promote the orientation of one-dimensional and two-dimensional fillers [28,29]. However, in the horizontal plane, one-dimensional fillers were still in a disordered arrangement [30]. In addition, the horizontally oriented structure of the fillers would lead to the highly anisotropic thermal conductivity of the composites, which could reach above 200 [31]. The thermal conductivity in the vertical direction was too low to satisfy the demand of heat dissipation, which was lower than 1.0 W/(m·K) [32]. Thus, preparing the composites with an excellent EMI shielding and thermal managed performance is still immensely challenging.

Many polymers have been used as a matrix to prepare the composites with a high thermal conductivity and EMI shielding performance [33]. Among the polymers that have been researched, polydimethylsiloxane (PDMS) has a good chemical resistance, it is highly electrical insulating, it has an easy processing, good weather resistance and thermal stability [34]. These excellent properties made PDMS widely used in 5G electronic equipment. In addition, the outstanding flexibility of PDMS is desirable for flexible electronics [35], such as wearable devices. Recently, the PDMS-based thermal conductive and EMI shielding composites have attracted great attention. For example, Gu et al. [36] prepared PDMS composites by using the cellulose carbon-reduced graphene oxide aerogels. The EMI shielding effectiveness of the composite reached 51 dB, while the thermal conductivity reached 0.65 W/(m·K). Lu et al. [37] constructed the bubble-templated graphene network through a foaming method and used it to prepare the PDMS composites with a high thermal conductivity and EMI shielding performance.

The synergistic effect of fillers with different shapes have been proven effective in improving the thermal conductivity of polymer composites [38]. For example, Yu et al. [39] used spherical alumina ang graphene as fillers and increased the thermal conductivity of epoxy composite to 13.3 W/(m·K). Zhang et al. [40] used alumina to bridge the vertically aligned carbon fiber for increasing the thermal conductivity of the PDMS composites. More importantly, the viscosity of the polymer was slightly increased with the addition of spherical alumina [41]. Thus, alumina was considered to be a good candidate for thermal conductive fillers.

In this work, short-cut carbon fibre (CF) was used as the conductive filler, due to its advantages of low-cost and easy processing, compared with graphene and carbon nanotubes [42]. We reported a strategy of combining the ice-templated assembly and stress-induced orientation to the prepared PDMS composites with a high orientation in the horizontal direction. Firstly, the aligned CF scaffolds were constructed by ice-templated assembly. Then, the scaffolds were backfilled with PDMS/alumina compounds. Lastly, the filled scaffolds were rotated 90 degrees and this was followed by a stress-induced orientation process. Alumina was used to connect with CF so that the continuous thermal conductive pathways were formed in both the horizontal and vertical directions. This work could provide a guidance for preparing polymer composites with a high thermal conductivity and EMI shielding effectiveness.

## 2. Materials and Methods

### 2.1. Materials

CF was purchased from Carbonene Technology (Shenzhen) Co., Ltd., Shenzhen, China. Spherical alumina (particle size 500 nm) was supplied by Shanghai Bestry Performance Materials Co., Ltd., Shanghai, China. Cellulose nanofiber was purchased from Zhongshan NanoFC Bio-materials Co., Ltd., Zhongshan, China. Sodium carboxymethyl cellulose (CMC, M.W. 90,000 (DS = 0.7), 50–100 mPa.s) was purchased from Shanghai Aladdin Bio-chem Technology Co. Ltd., Shanghai, China. 1-Ethynyl-1-cyclohexanol was purchased from Shanghai Macklin Biochemical Co., Ltd., Shanghai, China. PDMS and catalyst were obtained from Shanghai Guiyou New Material Technology Co., Ltd., Shanghai, China.

### 2.2. Preparation of the Aligned CF Scaffolds

CF and CMC (CF/CMC weight ratio was 100:2) were added to deionized water and sonicated for 10 min, followed by vigorous stirring for 2 h. Then, the cellulose nanofiber was added into the CF/CMC dispersion with further stirring for 2 h at room temperature. The concentration of the cellulose nanofiber was 10 mg/mL. Subsequently, the CF/CMC/cellulose nanofiber aqueous dispersion was poured into a Teflon mold. The bottom of the mold contacted the liquid nitrogen which was used as cryogen for forming a temperature gradient from bottom to top. The outside of the mold was enclosed with heat-insulation foam. The dispersion was frozen and then freeze dried at the low temperature of −50 °C and low pressure of 15 Pa for 48 h. Finally, the aligned CF scaffolds were successfully prepared.

### 2.3. Preparation of the Horizontally Oriented PDMS/Alumina/CF Composites

Firstly, PDMS, 1-ethynyl-1-cyclohexanol and alumina were mixed at room temperature to obtain the PDMS/alumina compounds. The concentration of 1-ethynyl-1-cyclohexanol was 0.1 wt%. Then, the CF scaffolds were placed in a Teflon mold. The PDMS/alumina compounds were poured into the mold, which was used to fill the CF scaffolds in a vacuum condition at room temperature for 4 h. The filled CF scaffolds were rotated 90 degrees and compressed, followed by vulcanization at a temperature of 120 °C in an oven. The compression ratio of the thickness of the CF scaffolds was about 5. Finally, the horizontally oriented PDMS/alumina/CF composites were obtained.

### 2.4. Characterization

The Fourier transform infrared (FTIR) spectra tests were conducted on a Nicolet iS20 FTIR Spectrometer (ThermoFisher Scientific, Waltham, MA, USA). The zeta potential was measured by a Malvern Nano ZS90 Zetasizer through the electrophoretic light scattering method. The morphology and microstructure of the CF, CF scaffolds and the PDMS/alumina/CF composites were observed with a FEI Nova Nano450 field emission scanning electron microscope (SEM). The mechanical properties of the composites were tested by an Instron 4465 machine. The tensile properties of the composites were tested, according to ASTM D412. The tear strength of the composites was tested, according to ASTM D624. The dumbbell-shaped and right-angle specimens were used in the tensile properties and tear strength tests, respectively. The thermal conductivities of the composites were measured with a Netzsch LFA467 laser flash analyzer using a laser flash method. The thermal conductivities in the vertical and horizontal directions were measured using the “through-plane” mode and “in-plane” mode, respectively. The electrical conductivity was measured with a FT-340 double electric four-probe resistance ratio tester. The EMI shielding effectiveness (SE) of the PDMS/alumina/CF composites was tested using a vector network analyzer (Anritsu MS4644A, Atsugi-shi, Japan) in the frequency range of 8.2–12.4 GHz through a waveguide method. The rectangular samples with dimensions of 22.5 × 10.0 × 3 mm^3^ were used for testing.

## 3. Results and Discussion

### 3.1. Preparation and Characterization of the Aligned CF Scaffolds

**Figure 1:**
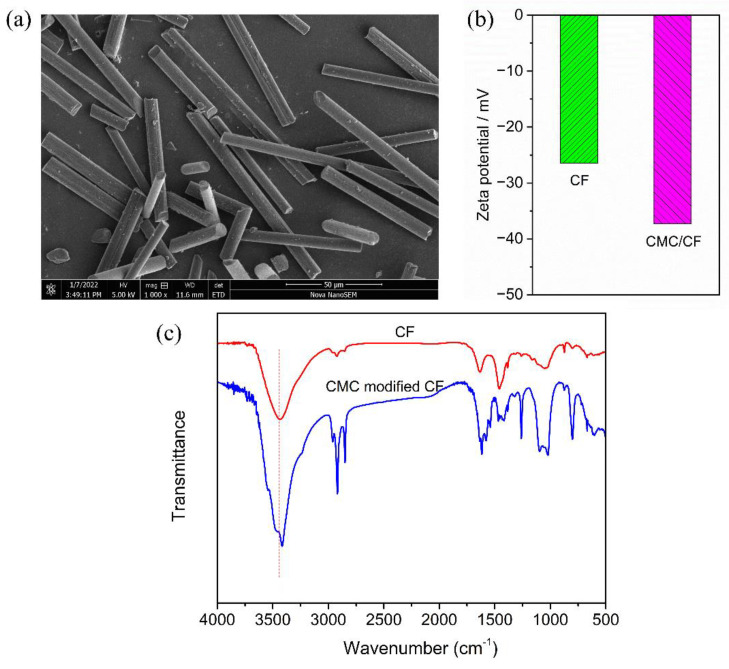
(**a**) SEM image of the CF, (**b**) Zeta potential of the CF and CMC/CF, (**c**) FTIR spectra of the CF and CMC/CF.

In this work, CF was used as the electrical and thermal conductive filler to improve the EMI shielding effectiveness and thermal conductivity of the PDMS composites. The morphology of the CF is shown in Figure 1a. The diameter and length of the CF were 4–9 μm and 50–150 μm, respectively, exhibiting a high aspect ratio. The high aspect ratio structure is beneficial to constructing the electrical and thermal conductive networks [43]. The CMC was used to improve the dispersion of the CF in deionized water. As shown in Figure 1b, the Zeta potential of CF was −26.5 mV, which can be attributed to the oxygen-containing groups on the surface of the CF. With the addition of CMC, the Zeta potential of CMC/CF reached −37.3 mV. The increased absolute value indicated that the electrostatic repulsion increased. The result proved that CMC could improve the dispersion stability of the CF in deionized water.

In order to illustrate the interfacial interaction, the FTIR spectra of the CF and CMC/CF were recorded and shown in Figure 1c. The CMC/CF was prepared through the process described below. Firstly, 0.1 g CF and 2 mg CMC were added into 1.0 mL deionized water under sonication for 10 min. Then, the dispersion was mechanically stirred for 2 h. Finally, CMC/CF was obtained through filtration, washing and drying. As shown in Figure 1c, the absorption peak of the CF at 3439 cm^−1^ was attributed to the stretching vibration of hydroxyl (-OH) on the surface of the CF [44]. The absorption peak of the CF at 1632 cm^−1^ corresponded to the stretching vibration peak of carbonyl (C=O) on the surface of the CF. The oxygen-containing groups on the surface of the CF could interact with CMC through the hydrogen bonds. The absorption peaks of CMC/CF at 3416 cm^−1^, 1616 cm^−1^, 1260 cm^−1^ and 1022 cm^−1^ corresponded to hydroxyl (-OH), carbonyl (C=O), C–O–H and C–O–C, respectively. The absorption peaks of CMC/CF at 2959 cm^−1^, 2917 cm^−1^, and 2849 cm^−1^ were assigned to methyl (-CH_3_) and methylene (-CH_2_-), respectively [45]. The red-shifting of the -OH and C=O vibration bands of CMC/CF, resulted from the hydrogen bonding between CMC and CF.

**Figure 2:**
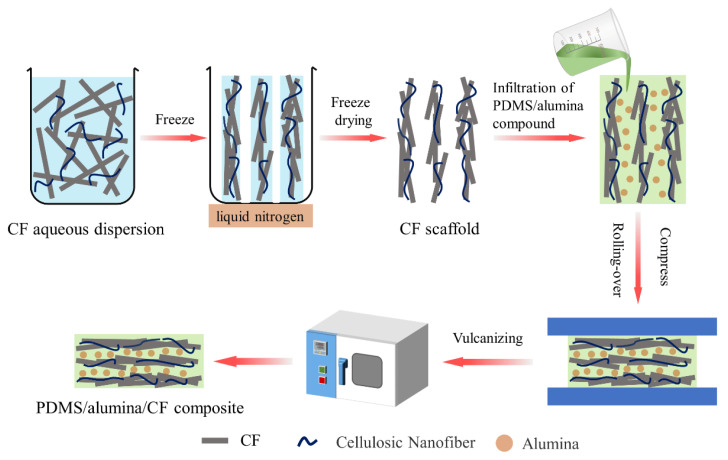
Schematic diagram of the preparation of the CF scaffolds and PDMS/alumina/CF composites.

**Figure 3:**
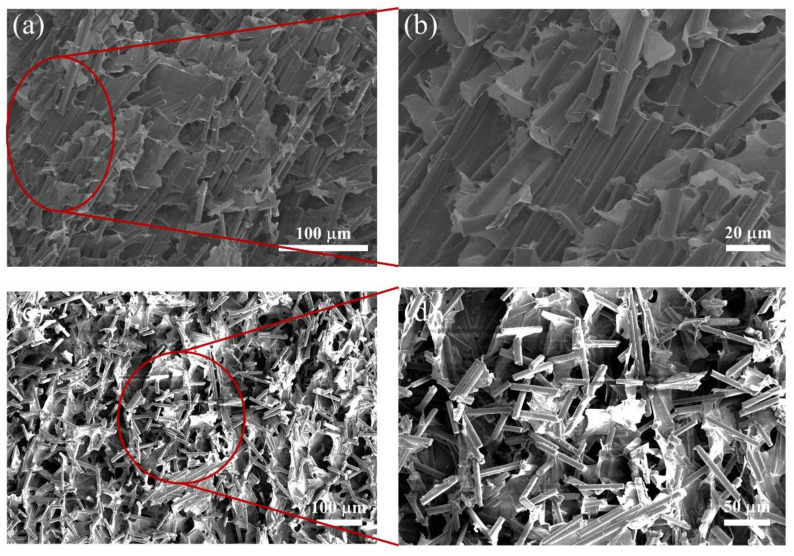
(**a**,**b**) Fracture surface SEM images of the CF scaffolds with the addition of CMC, (**c**,**d**) Fracture surface SEM images of the CF scaffolds without CMC.

In order to improve the orientation degree of the CF, the ice-templated assembly was utilized for constructing the aligned CF scaffolds, as shown in Figure 2. During the preparation of the CF scaffolds, the bottom of the CF/CMC/cellulose nanofiber dispersion contacted with liquid nitrogen which could control the temperature. The obvious temperature gradient from bottom to top was formed. In the freeze casting, the ice crystals grew along the temperature gradient. The CF, CMC and cellulose nanofiber were squeezed out to the edge of the ice crystals. During this process, the CF were arranged regularly. Following the freeze-drying, the aligned CF scaffolds were obtained. The CMC and cellulose nanofiber acted as adhesives to link the CF. The microstructure images of the CF scaffolds are shown in Figure 3. Figure 3a,b exhibit the SEM images of the CF scaffolds with the addition of CMC. The CF scaffolds showed the structure with a highly ordered arrangement along the frozen direction. The highly ordered structure plays an important role in improving the EMI shielding effectiveness and thermal conductivity of the PDMS composites, due to the formation the electrical and thermal conductive pathways. Figure 3c,d exhibit the SEM images of the CF scaffolds without CMC. The disorganized structure of the CF scaffolds could be clearly observed. Without the addition of CMC, the CF exhibited the poor dispersibility in deionized water. In freeze casting, the agglomerated CF was trapped in ice crystals, due to the weak mobility. Thus, the CF scaffolds without CMC were disordered, which has negative effects on the EMI shielding effectiveness and thermal conductivity.

### 3.2. Preparation and Characterization of the Horizontally Oriented PDMS/Alumina/CF Composites

In order to further improve the orientation degree of the CF and perfect the electrical and thermal conductive pathways, the CF scaffolds were further processed by following these steps. Firstly, alumina and PDMS were mixed to prepare PDMS/alumina compounds. Then, the compounds were filled in the CF scaffolds by the vacuum-assisted impregnation. Prior to the vulcanization of PDMS, the CF scaffolds filled with the PDMS/alumina compounds were rotated 90 degrees and compressed, as shown in Figure 2. During the compression process, the CF was rearranged through the stress-induced orientation. Finally, the horizontally oriented PDMS/alumina/CF composites were prepared successfully after the vulcanization of PDMS.

**Figure 4:**
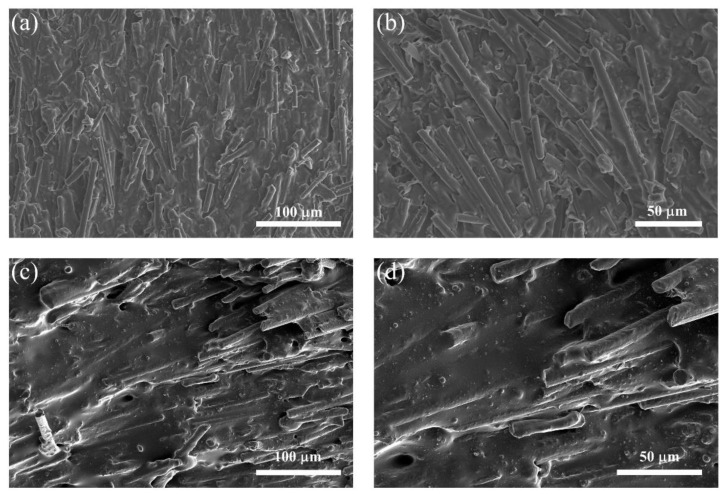
(**a**,**b**) Fracture surface SEM images of the PDMS/CF composite without alumina, (**c**,**d**) Fracture surface SEM images of the PDMS/alumina/CF composite.

The microstructure images of the composites are shown in Figure 4. Figure 4a,b exhibit the SEM images of the PDMS/CF composite without alumina. The highly oriented CF in the horizontal direction could be observed in the PDMS/CF composite. The high orientation degree could be attributed to the comprehensive effect of the ice-templated assembly and stress-induced orientation. The SEM images of the PDMS/alumina/CF composite are shown in Figure 4c,d. The highly oriented CF could also be observed in the PDMS/alumina/CF composite. In addition, alumina was randomly distributed in the PDMS matrix. The alumina was filled in the space among CFs and was connected to CF, which contributed to the formation of the thermal conductive pathways in both the horizontal and vertical directions. The addition of alumina could alleviate the impedance mismatching due to the high electrical insulation of alumina.

**Figure 5:**
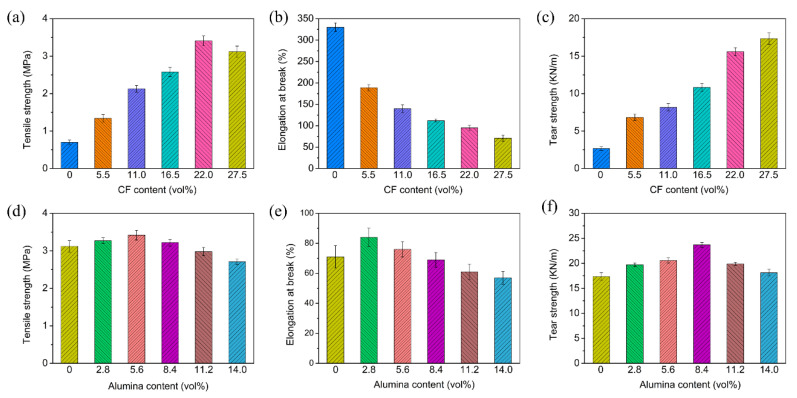
(**a**–**c**) The tensile strength, elongation at the break and tear strength of the PDMS/CF composites, respectively, (**d**–**f**) The tensile strength, elongation at the break and the tear strength of the PDMS/alumina/CF composites, respectively.

**Figure 6:**
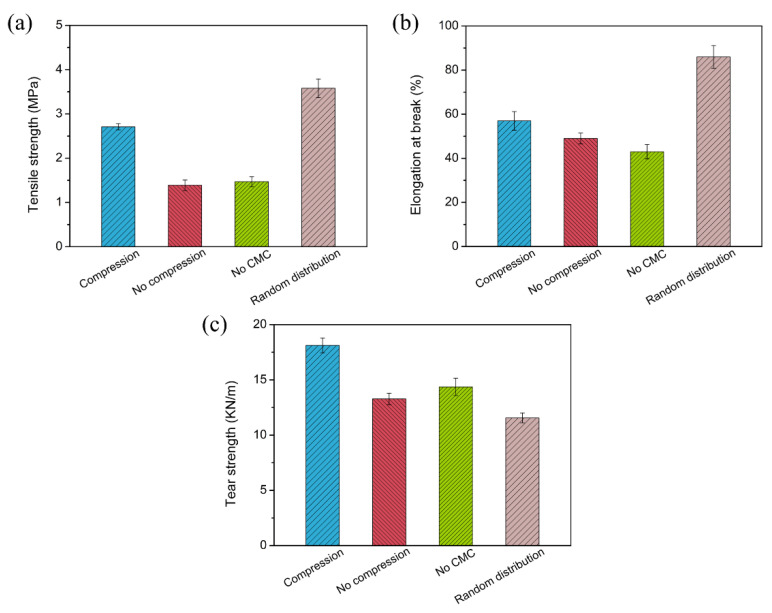
Comparisons of the mechanical properties of the composites at the same content of 27.5 vol% CF and 14.0 vol% alumina: (**a**) tensile strength, (**b**) elongation at break and (**c**) tear strength.

The mechanical properties of the PDMS/CF and PDMS/alumina/CF composites were measured. The tensile strength and elongation at the break of the PDMS/CF composites are shown in Figure 5a,b. The tensile strength of the composites increased with the increasing CF content from 0 to 22.0 vol%. With 22.0 vol% of the CF incorporated into PDMS, the tensile strength increased from 0.70 MPa to 3.41 MPa. The enhancement of the tensile strength could be attributed to the reinforcing effect of the CF on PDMS. However, when the CF content increased from 22.0 vol% to 27.5 vol%, the tensile strength of the composites decreased from 3.41 MPa to 3.12 MPa, caused by the stress concentration at a high filler content. The elongation at the break of the PDMS/CF composites decreased with the increasing CF content. The elongation at the break of PDMS was 330%. Following the addition of 27.5 vol% of CF, the elongation at the break of the composite decreased to 71%. The dependence of the tear strength of the PDMS/CF composites on the CF content is shown in Figure 5c. The tear strength of the PDMS/CF composites increased significantly with the increasing CF content. With the addition of 27.5 vol% CF, the tear strength increased to 17.4 KN/m, which was about 6.5 times, compared with that of PDMS. The high tear strength was caused by the high aspect ratio and the highly oriented structure of the CF which could suppress the crack propagation. The dependence of the tensile strength and the elongation at the break of the PDMS/alumina/CF composites at the CF content of 27.5 vol% on the alumina content, are shown in Figure 5d,e, respectively. The tensile strength of the PDMS/alumina/CF composites increased firstly and then decreased with the increasing alumina content. The tensile strength of the composite, a maximum of 3.42 MPa, with the alumina content of 5.60 vol%. The decrease of the tensile strength of the high alumina content was mainly due to the agglomeration of alumina. The elongation at the break of the PDMS/alumina/CF composites also increased firstly and then decreased with the increasing alumina content. The dependence of the tear strength of the PDMS/alumina/CF composites with a CF content of 27.5 vol% on the alumina content is shown in Figure 5f. As the alumina content increased from 0 to 8.40 vol%, the tear strength of the composites increased from 17.4 KN/m to 23.7 KN/m. When the alumina content further increased to 14.0 vol%, the tear strength of the composites decreased to 18.1 KN/m, which was still higher than that of the composite without alumina. Comparisons of the mechanical properties of the PDMS/alumina/CF composite, the composite without compression, the composite without CMC and the composite with the randomly distributed filler and at the same filler content of 27.5 vol% CF with 14.0 vol% alumina, are shown in Figure 6. As shown in Figure 6a,b, the composite with the randomly distributed filler had the highest tensile strength and elongation at the break, which could be attributed to the reduction of the filler agglomeration, compared with the oriented structure. The composite without compression and the composite without CMC exhibited poor mechanical properties, due to the serious filler agglomeration. As shown in Figure 6c, the PDMS/alumina/CF composite had the highest tear strength, due to the highest orientation degree.

### 3.3. Thermal Conductivities of the Horizontally Oriented PDMS/Alumina/CF Composites

**Figure 7:**
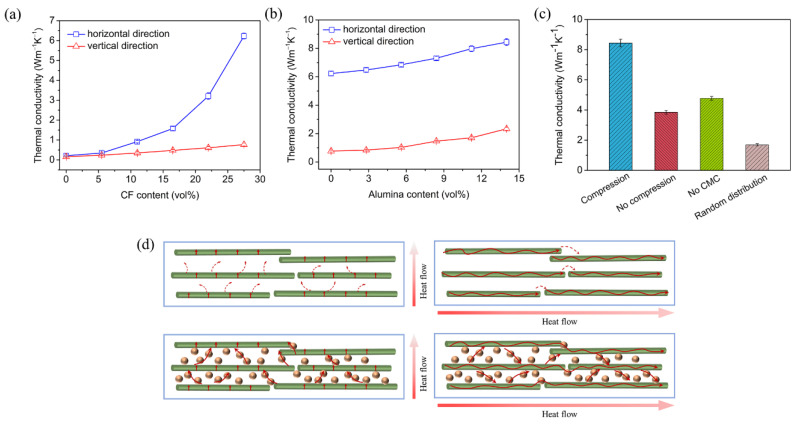
(**a**) The dependence of the thermal conductivities of the PDMS/CF composites in the horizontal and vertical directions on the CF content when the alumina content was 0, (**b**) the dependence of the thermal conductivities of the PDMS/alumina/CF composites in the horizontal and vertical directions on the alumina content when the CF content was 27.5 vol%, (**c**) a comparison of the thermal conductivities in the horizontal direction of the PDMS/alumina/CF composite, the composite without compression, the composite without CMC and the composite with a randomly distributed filler at the same content of 27.5 vol% CF and 14.0 vol% alumina, (**d**) the thermal conduction mechanisms of the PDMS/CF and PDMS/alumina/CF composites.

The dependence of the thermal conductivities in the horizontal and vertical directions on the CF content is shown in Figure 7a. The thermal conductivity of the composite in the horizontal direction increased significantly with the increasing CF content. The thermal conductivity of PDMS was only 0.21 W/(m·K). With 27.5 vol% of the CF incorporated into PDMS, the thermal conductivity in the horizontal direction reached 6.23 W/(m·K), corresponding to 29.7 times higher than that of PDMS. The thermal conductivity of the composite in the vertical direction increased slowly with the increasing CF content. The thermal conductivity of the composite with 27.5 vol% CF in the vertical direction was 0.77 W/(m·K). The horizontally oriented PDMS/CF composites exhibited a significant anisotropy in the thermal conductivity. The dependence of the thermal conductivities of the PDMS/alumina/CF composites in the horizontal and vertical direction on the alumina content is shown in Figure 7b. Both thermal conductivities in the horizontal and vertical direction increased with the increasing alumina content. The thermal conductivity in the horizontal direction increased from 6.23 to 8.44 W/(m·K) when the alumina content increased from 0 to 14.0 vol%, while the thermal conductivity in the vertical direction increased from 0.77 to 2.34 W/(m·K). The thermal conductivity in the horizontal direction was 40.2 times higher than that of PDMS. The enhancement of the thermal conductivity illustrated that the bridging effect of alumina promoted the connectivity of the thermal conductive pathways in both the horizontal and vertical directions. A comparison of the thermal conductivities of the PDMS/alumina/CF composite, the composite without compression, the composite without CMC and the composite with randomly distributed filler is shown in Figure 7c. The thermal conductivity of the PDMS/alumina/CF composite was 5.0 times higher than that of the composite with the randomly distributed filler. The composite with the randomly distributed filler exhibited the lowest thermal conductivity, due to the few connections among the CF and alumina. The PDMS/alumina/CF composite had the highest thermal conductivity, due to the highly oriented structure and the multitudinous thermal conductive pathways. The thermal conduction mechanisms of the PDMS/CF and PDMS/alumina/CF composites are shown in Figure 7d. As shown in the schematic diagram of the thermal conduction of the PDMS/CF composite, there are the gaps between the adjacent CF in the vertical direction which led to the high interfacial thermal resistance, due to the phonon scattering. Thus, the thermal conductivity of the PDMS/CF composite in the vertical direction was low. For the thermal conduction in the horizontal direction, the heat was transported unidirectionally along the aligned CF, which led to a high thermal conductivity. However, the gaps between the adjacent CF in the horizontal direction restricted the enhancement of the thermal conductivity. As shown in the schematic diagram of the thermal conduction of the PDMS/alumina/CF composite, alumina was filled in the gaps between the adjacent CF, contributing to the formation of continuous thermal conductive pathways, which reduced the interfacial thermal resistance. Thus, both thermal conductivities in the horizontal and vertical directions were significantly increased.

### 3.4. Electrical Conductivities and Electromagnetic Shielding Performance of the Horizontally Oriented PDMS/Alumina/CF Composites

**Figure 8:**
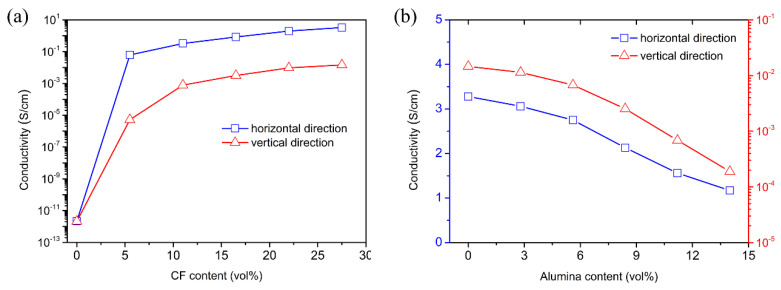
(**a**) The dependence of the electrical conductivities of the PDMS/CF composites in the horizontal and vertical directions on the CF content when the alumina content was 0, (**b**) the dependence of the electrical conductivities of the PDMS/alumina/CF composites in the horizontal and vertical directions on the alumina content when the CF content was 27.5 vol%.

Figure 8a shows the electrical conductivities of the PDMS/CF composites as a function of the CF content. The electrical conductivities in the horizontal and vertical directions increased significantly with the increasing CF content, due to the high conductivity of CF. With the addition of 27.5 vol% CF, the electrical conductivity of the composite in the horizontal direction reached 3.28 S/cm, while that in the vertical direction was 1.46 × 10^−2^ S/cm. The high anisotropy in the electrical conductivity was caused by the horizontally oriented structure. Figure 8b shows the electrical conductivities of the PDMS/alumina/CF composites with 27.5 vol% CF as a function of the alumina content. The electrical conductivity in the horizontal direction decreased slightly with the increasing alumina content. When the alumina content was 14.0 vol%, the electrical conductivity in the vertical direction was decreased by two orders of magnitude. With the addition of alumina, the electrically conductive pathways in the vertical direction were obstructed, as a result of the high electrical insulation of alumina. The decrease of the electrical conductivity could reduce the impedance mismatch between the PDMS/alumina/CF composite and the air interfaces.

**Figure 9:**
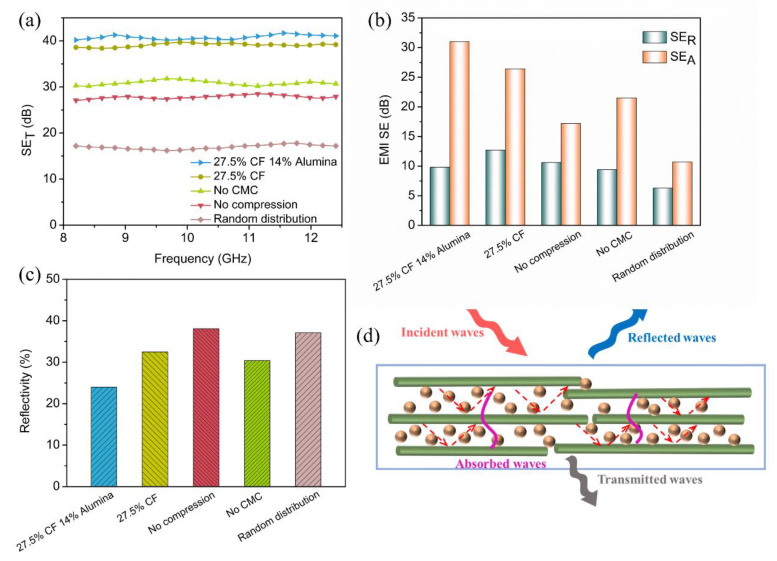
EMI shielding performance of the PDMS/alumina/CF composite, the PDMS/CF composite, the composite without compression, the composite without CMC and the composite with the randomly distributed filler: (**a**) SE_T_ over the X-band frequency ranges, (**b**) SE_A_ and SE_R_, (**c**) reflectivity and (**d**) EMI shielding mechanism of the PDMS/alumina/CF composite.

Figure 9a shows the total EMI shielding effectiveness (EMI SE_T_) values of the PDMS/alumina/CF composite, the PDMS/CF composite, the composite without compression, the composite without CMC and the composite with the randomly distributed filler over the X-band frequency ranges (8.2–12.4 GHz). As shown in Figure 9a, the PDMS/alumina/CF composite with 27.5 vol% CF and 14.0 vol% alumina had a highest average SE_T_ value, which was 40.8 dB. The PDMS/CF composite with 27.5 vol% CF also exhibited a good EMI shielding performance whose average SE_T_ value was 39.1 dB. The results indicated that the CF played a main role in improving the EMI shielding performance of the composites. The average SE_T_ values of the composite without compression and the composite without the CMC were 27.8 dB and 30.9 dB, respectively. The composite with the randomly distributed filler exhibited a lowest average SE_T_ value of 17.0 dB. These results indicated that the highly oriented structure had a positive effect on the improvement of the EMI shielding performance. According to the Schelkunoff theory [46], SE_T_ consists of the absorption (SE_A_), reflection (SE_R_), and the multiple reflections shielding effectiveness (SE_M_). Commonly, SE_M_ could be omitted when SE_T_ is higher than 10 dB.

As shown in Figure 9b, the SE_A_ of all composites were higher than SE_R_, indicating the shielding effectiveness of the absorption was dominant in the SE_T_. Compared with the PDMS/CF composite, the PDMS/alumina/CF composite had a lower SE_R_ and a higher SE_A_. The SE_A_ of the PDMS/alumina/CF composite was much higher than that of the composite without CMC. The reflectivity was defined as the ratio of SE_R_ to SE_A_, which was shown in Figure 9c. The PDMS/alumina/CF composite exhibited a lowest reflectivity of 24.0%, while the reflectivity of the other composites was higher than 30.0%. The decrease in reflectivity could reduce the secondary pollution of the electromagnetic waves. The EMI shielding mechanism of the PDMS/alumina/CF composite was illustrated in Figure 9d. When the incident electromagnetic waves reached the surface of the PDMS/alumina/CF composite, a part of the electromagnetic waves were immediately reflected, due to the impedance mismatching. Owing to the high electrical insulation of alumina, the content of the reflected electromagnetic waves was reduced. The remaining electromagnetic waves transmitted into the composite. The electromagnetic waves interacted with the electron in the CF, leading to ohmic losses which dissipated the energy of the electromagnetic waves. More importantly, multiple reflections of electromagnetic waves occurred between the adjacent CF with a highly oriented structure, thereby leading the electromagnetic waves to be absorbed many times. Thus, the increase of the SE_A_ was not only due to the high electrical conductivity, but also to the highly oriented structure of the CF. These results illustrated that the horizontally oriented PDMS/alumina/CF composites exhibited an excellent EMI shielding performance.

## 4. Conclusions

In this work, the horizontally oriented PDMS/alumina/CF composites were successfully prepared by the ice-templated assembly, followed by the stress-induced orientation. The highly oriented structure of the PDMS/alumina/CF composites was confirmed by the morphology characterization. The composites exhibited good mechanical properties, due to the reinforcing effects of the CF and alumina, especially a high tear strength due to the oriented structure. The horizontally oriented CF provided the electrically and thermally conductive pathways in the horizontal direction. The addition of alumina contributed to the formation of thermal conductive pathways in both the horizontal and vertical directions. The thermal conductivities of the PDMS/alumina/CF composite with the CF content of 27.5 vol% and the alumina content of 14.0 vol% in the horizontal and vertical directions reached 8.44 and 2.34 W/(m·K), respectively, exhibiting excellent thermal conductive properties. The enhancement of the thermal conductivity was attributed to the oriented structure of the CF and the bridging effect of alumina. In addition, the composite exhibited a high EMI shielding effectiveness of 40.8 dB and a low reflectivity of 24.0%. This work could give an inspiration for the development of polymer composites with an excellent thermal conductivity and EMI shielding performance.

## Data Availability

Not applicable.
